# Carbon Nanotubes Dispersion Assessment in Nanocomposites by Means of a Pulsed Thermographic Approach

**DOI:** 10.3390/ma13245649

**Published:** 2020-12-11

**Authors:** Nicola Montinaro, Mario Fustaino, Antonio Pantano

**Affiliations:** Dipartimento di Ingegneria, Università degli Studi di Palermo, 90128 Palermo, Italy; nicola.montinaro@unipa.it (N.M.); mario.fustaino@unipa.it (M.F.)

**Keywords:** nanocomposite, carbon nanotube, NDE, thermographic inspection, IR-NDT, PPT

## Abstract

The extensive production of polymer composites reinforced by carbon nanotube is limited by the absence of non-destructive evaluation (NDE) methods capable of assessing product quality to guarantee compliance with specifications. It is well known that the level of dispersion of carbon nanotubes (CNTs) in the polymer matrix is the parameter that, much more than others, can influence their enhancement capabilities. Here an active Infrared Thermography Non Destructive Testing(IR-NDT) inspection, joined with pulsed phase thermography (PPT), were applied for the first time to epoxy-CNT composites to evaluate the level of dispersion of the nanoparticles. The PPT approach was tested on three groups of epoxy nanocomposite samples with different levels of dispersion of the nanoparticles. The phasegrams obtained with the presented technique clearly show clusters, or bundles, of CNTs when present, so a comparison with the reference sample is not necessary to evaluate the quality of the dispersion. Therefore, the new NDE approach can be applied to verify that the expected dispersion levels are met in products made from epoxy and Multi-Walled Carbon Nanotubes (MWCNTs). The mechanisms underlying the effects of the dispersion of carbon nanotube on the thermal response of polymer composites have been identified.

## 1. Introduction

Carbon nanotubes (CNTs) have exceptional mechanical properties, which combined with high geometrical aspect, stiffness to weight and strength to weight ratios, give them the potential to be the ideal reinforcing agents in composites e.g., [1,2,3,4,5,6,7,8,9]. Nevertheless, by adding CNTs, thermal e.g., [10,11,12,13,14], electrical e.g., [15,16,17,18], and optical properties e.g., [19,20,21,22] of formed composites can also be improved. These high potentials collide with some issues that considerably hinder the use of polymeric composites enhanced by CNTs. One of the most important is the absence of non-destructive evaluation (NDE) techniques able to easily, and at low cost, control the quality of the products made, in order to ensure compliance with their specifications. It is well known that the level of dispersion of CNTs in the polymer matrix is the parameter that, much more than others, can influence their enhancement capabilities [23,24,25]. The poor dispersion and formation of bundles significantly limit the enhancements achievable by adding CNTs to the neat polymer. An NDE technique based on the impulse acoustic microscopy method, and that was able to control the quality of the graphene dispersion has been presented in literature [26,27]. They were used to control bulk microstructure in carbon nanocomposite samples with different levels of dispersion; however, this NDE method is slow and can only scan small samples, thus limiting possible industrial applications. In [28], a technique based on dynamic scanning calorimetry (DSC) measurement data was proposed to represent the degree of CNTs dispersion. The model indicated that the exothermic reaction heat during the curing process is a good quantitative measure to estimate the mechanical property of nanocomposites, as well as to evaluate the degree of CNTs dispersion.

In a previous work [29], we presented a new non-destructive technique able to determine the dispersion of CNTs in composites, based on infrared thermography. Nanocomposite samples with different levels of nanoparticle dispersion were manufactured. Several pairs of samples were compared, and it was found that there was a distinct difference in their thermal response. The method, however, was born with a crucial limitation—it works only as a comparison between pairs of specimens side by side, examined simultaneously by an IR camera. Furthermore, the only difference between the two specimens must be the dispersion; there must be no other difference, either in terms of size or in terms of composition.

To overcome this limitation, here an active infrared Infrared Thermography Non Destructive Testing (IR-NDT) inspection technique was applied for the first time to epoxy-CNTs composites to evaluate the level of dispersion of the nanoparticles. Infrared-NDT techniques (IR-NDT) allow, with flexible hardware setups, a fast full-field non-contact inspection of large areas. When a sample surface is pulse heated, the radiation generates a series of thermal waves with different amplitudes and frequencies that propagates inside the medium in a transient mode. It is known that the pulse can be decomposed into a multitude of individual sinusoidal components, and that, using the Fourier transform tool, itis possible to link temporal and frequency domains. In a pulsed phase thermography (PPT) approach, the acquisition procedure of “pulsed thermography” (PT) is combined with the phase and frequency concepts of “lock-in thermography” (LT), for which specimens are submitted to a periodical excitation [30,31,32]. A heat pulse of high intensity and low duration is generated by flash lamps, and the following temperature decay is then acquired over a truncation window [33,34]. Once raw data are collected, the discrete Fourier transform is calculated to evaluate the frequency content of the thermal response. The phase of the low-frequency harmonic terms is finally obtained and presented in the form of phasegrams, with local phase contrast representing a potential issue in the sample structures (e.g., inhomogeneity, cluster of CNT bundles). In the PPT approach, it is expected that deeper anomalies are better contrasted in low frequency phasegrams, while higher frequency phasegrams probe better for superficial issues. The signal normalization inherent in the evaluation of the phase is also expected to reduce the counter effects of non-uniform heat deposition and environmental reflections, similarly to PPT and LT [35].

In this work, the pulsed thermography setup is applied to test three groups of epoxy nanocomposites samples with dissimilar degrees of nanoparticle dispersion, manufactured with different procedures. The outcome of the PT is analyzed with the pulsed phase thermography (PPT) approach and the resulting phasegrams are used to evaluate the dispersion. The phasegrams clearly show clusters, or bundles, of CNTs when present, so a comparison with the reference sample is not necessary to evaluate the quality of the dispersion, as was necessary for the previous approach.

## 2. Materials and Methods

### 2.1. Preparation of the Epoxy-CNTs Samples

Three types of epoxy-CNTs samples (A, B, and C) were manufactured to achieve different levels of CNTs dispersion (from the coarse dispersion of the A batch to the finer dispersion of the C batch). Three batches were produced for type A and C, and one for the type B, for a total of 7 different batches. Then, 4 samples were obtained from each batch, for a total of 28 samples, which were all tested with the proposed technique.

**Figure 1:**
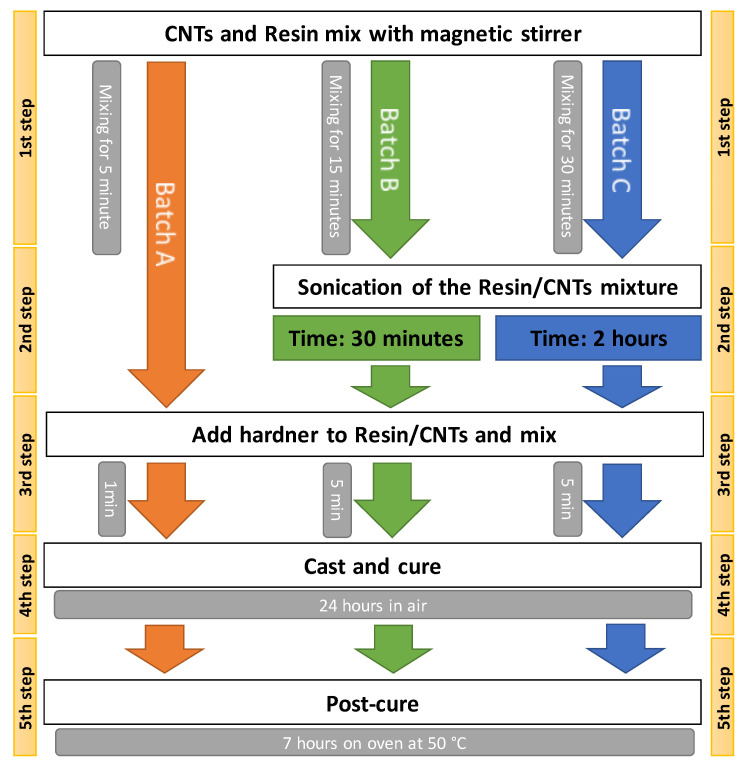
Block diagram of the manufacturing processes of the three batches of samples with different levels of CNTs dispersion.

In Figure 1, a schematic block diagram summarizes the production process of the three types of batches (A, B, and C). The bi-component epoxy resin, used as a matrix for the CNTs, is the “MATES SX8 EVO” while the CNTs are of the multi-wall type. The properties of the resin, hardener, and CNTs are listed in Table 1.

The manufacturing process of Figure 1 consists of five steps, where some specific variations are induced to obtain different degrees of CNTs dispersion. In the first step, a magnetic stirrer, model Arex (Velp Scientifica, Usmate, Italy), is used to mix the CNTs with the resin (without hardener) for about 5 min for the Batch A, 15 min for Batch B, and 30 min for Batch C. The next step is the dispersion of the CNTs bundles inside the resin-CNTs mixture in a sonication bath (with a power of 50 W and a frequency 40 kHz). This sonication step was skipped for the first A batch in order to obtain a coarse level of CNTs dispersion within the matrix. It is known how the sonication time influences the dispersion of particles e.g., [36]. In the third step the hardener is added to the resin-CNTs and homogenized with a magnetic stirrer for about 5 min for all three batches. In step four, the mixture is poured into a mold to obtain samples of the same dimensions (48 mm × 45 mm, with a thickness of 5 mm) and weight (11.4 ± 1 g); afterward the Epoxy-CNTs composites were cured for 24 h at room temperature (about 25 °C). In the last step, the solidified samples are removed from the mold and then post-cured in an oven, model L40/11/P320 (Nabertherm GmbH, Lilienthal, Germany), at 50 °C for 7 h.

**Figure 2:**
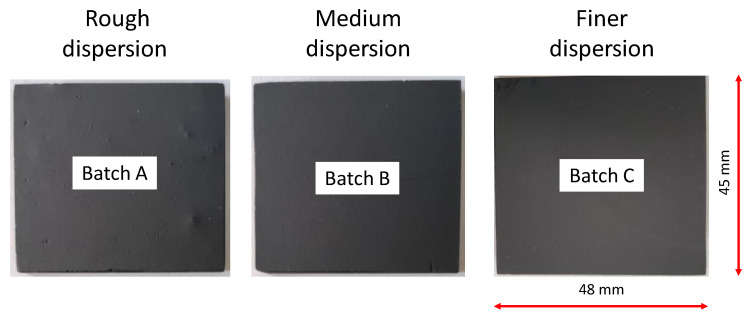
Image of three samples from each batch after applying the matte black paint.

Before the thermographic analysis, to enhance and make uniform the surface emissivity, two coats of a matt black paint were applied to the sample surfaces. Figure 2 shows an image of three samples from each batch after the application of the matt black paint. The thermal properties of the materials are listed in Table 2.

### 2.2. Pulsed Thermography Setup

**Figure 3:**
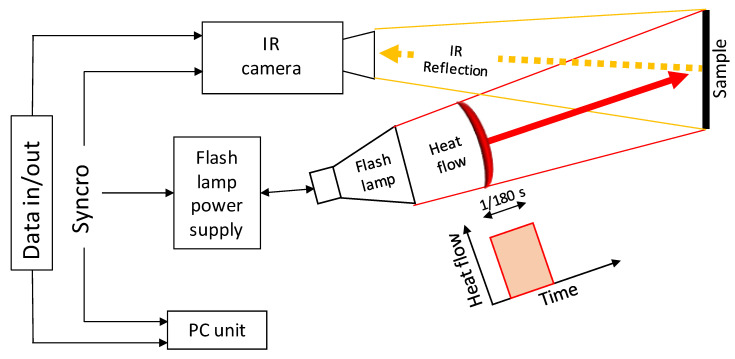
Schematic representation of the Pulsed Thermography experimental setup.

In Figure 3, the schematic representation of the PT experimental setup is shown. The flash lamp is placed on the same side as the infrared (IR) camera in reflection setup and provides a heat pulse of 4800 W/sec, see Table 3 for specifications. The IR-camera is focused on the sample surface and captures the full-field temperature evolution during the test performed. In Table 3, the main setup parameters of the PT test are shown.

In the PT test, the IR frame acquisition is triggered by a single pulse of the flash lamp. After the pulse, sampling is carried out within a time window, truncated by manually stopping data acquisition once the sample surface temperature becomes uniform. 

In this work, the experiments were performed using an InSb cooled IR camera (FLIR Systems, Wilsonville, OR, USA), FLIR X6540sc, equipped with a 25 mm F/2.0 lens (with field of view H × V = 21.74° × 17.46°). The heat source is an Elinchrom Flash lamp with a pulse of 4800 W/sec powered by a couple of Elinchrom 2400 rx in a parallel configuration.

## 3. Results

In the PT technique, the single heat pulse generates a series of thermal waves into the sample at different frequencies. The thermal response from the sample surface, acquired during the cooling transient, is analyzed in the frequency domain by the Discrete Fourier Transform (DFT), and the phases of the harmonic content retrieved from each pixel and shown in graphical form as a phasegrams image. 

**Figure 4:**
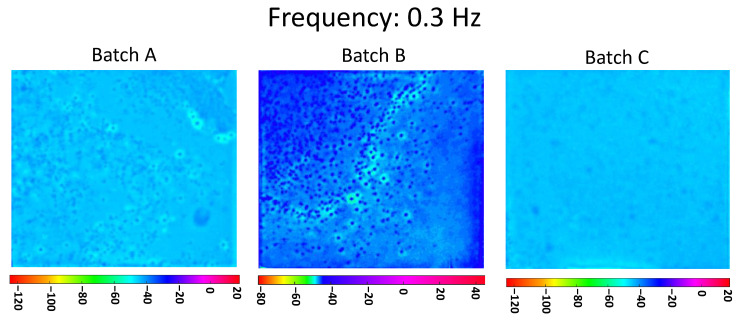
Pulsed phase thermography (PPT) phasegrams of a sample of each batch computed at the selected frequency 0.3 Hz.

Figure 4 shows the phasegrams for the three batches A, B, and C calculated at the selected frequency of 0.33 Hz (low frequency). As discussed earlier, in the PPT algorithm at a lower frequency corresponds with a deeper probing distance from the surface. Looking at Figure 4, it is possible to clearly distinguish the different levels of dispersion of nanoparticles between the three manufacturing processes, in agreement with the previsions. As will be discussed in Chapter 4, this behavior is due to the dissimilar thermal properties between the individual CNTs, the CNTs bundles, and the epoxy resin, as shown in Table 2.

Unlike the algorithms that map the thermal contrast from the amplitude of the temperature, the use of phasegrams prevents influences arising from surface emissivity. The latter statement is enforced by the application of the matt black paint on the sample surface, as described in the previous section. Figure 4 does not seem to show a clearly different homogenization level between Batch A and B, while in Batch C there is a clear increase in the homogeneity. The resolution of the technique with the setup shown in Table 3 is equal to 0.18 mm/px.

**Figure 5:**
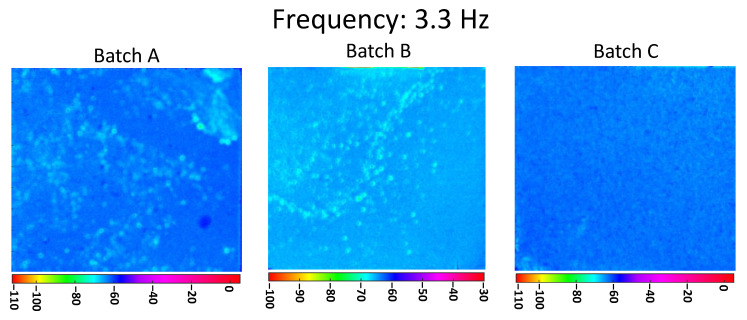
PPT phasegrams of a sample of each batch computed at the selected frequency 3.3 Hz.

In Figure 5, the phasegrams for the three batches, A, B, and C, calculated at the selected frequency of 3.3 Hz (high frequency), are shown. In the PPT algorithm, the investigation at higher frequency corresponds to a shorter distance from the inspected surface. Still, here in Figure 5, there is a clear difference between the dispersion of the nanoparticles on the three batches, which results in agreement with the corresponding manufacturing processes.

Comparing the results for Batch C reported in Figure 4 and Figure 5, it can be seen how the modest inhomogeneity reported in Figure 4 have almost completely disappeared in Figure 5, proving that the dispersion of the clusters inside the sample is not yet optimal. The same comparison for Batch A and Batch B shows the inhomogeneity is still visible with a superficial scan (Figure 5) which demonstrates that CNTs clusters are located throughout the sample thickness.

It is therefore remarkable how the results of Figure 4 and Figure 5 show a negligible increase in the CNTs dispersion between Batch A and Batch B, thus indicating that the most relevant contribution to homogenize the particles is sonication (the second step in the manufacturing process).

In order to validate the robustness of the thermographic approach in the detection of CNTs clusters, a second and a third Batch A and Batch C, from here on A/2, C/2, A/3, C/3, were manufactured and then tested.

**Figure 6:**
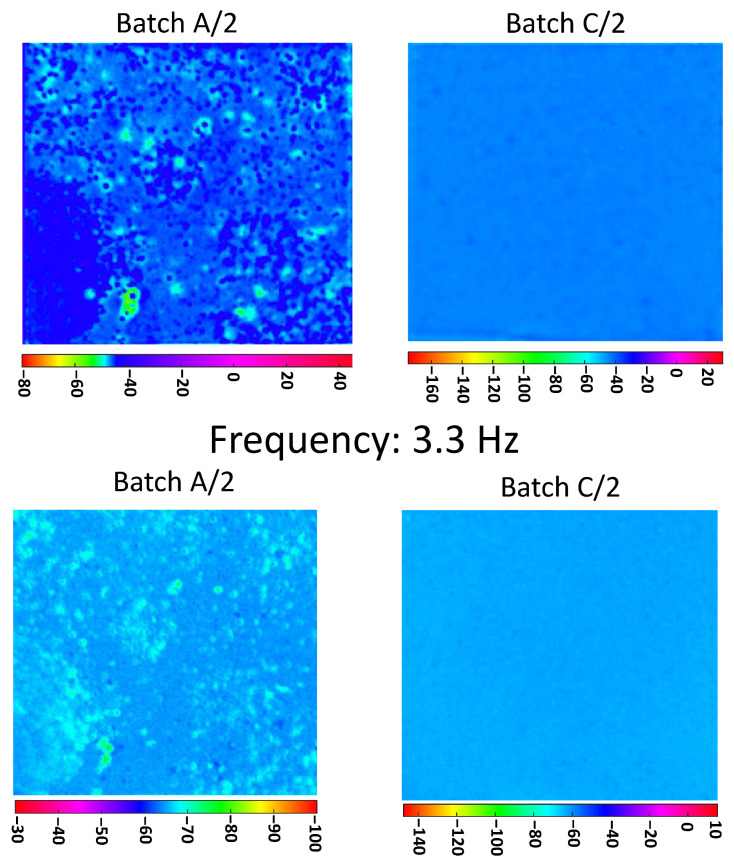
PPT phasegrams comparison of samples coming from A/2 and C/2; on the first row phasegrams computed at selected frequency of 0.3 Hz, on the second row phasegrams computed at selected frequency 3.3 Hz.

In Figure 6, the phasegrams, relative to the two batches A/2 and C/2, show the effectiveness of the technique in retrieving the dispersion level of the nanoparticle for shallow (3.3 Hz) and deep probing distance (0.3 Hz). The results confirm the outcomes from the first batches (A, B, and C).

**Figure 7:**
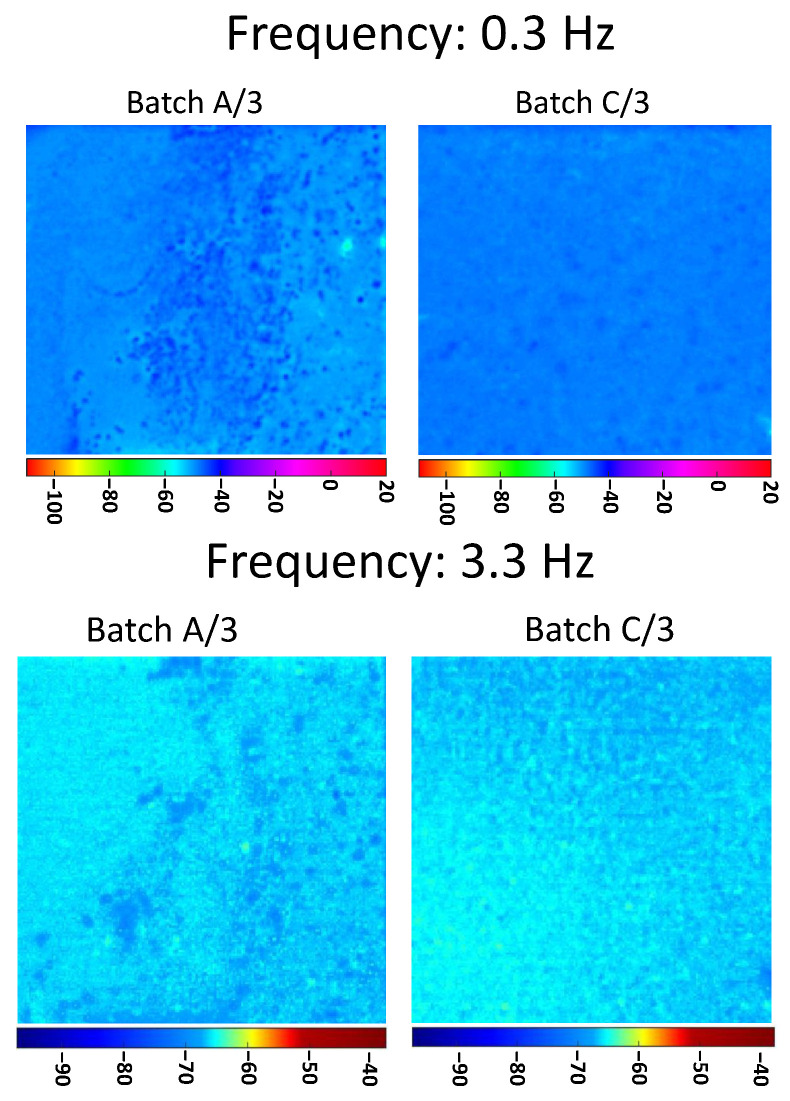
PPT phasegrams comparison of samples coming from A/3 and C/3; on the first row phasegrams computed at selected frequency of 0.3 Hz, on the second row phasegrams computed at selected frequency 3.3 Hz.

In Figure 7, the phasegrams, relative to the two batches A/3 and C/3, further validate the proposed method.

To investigate the inner structure of the epoxy-CNTs composite, a series of cross-sectional macrographs of a sample from Batch A and of a sample from Batch C were captured. A Nikon D5100 camera equipped with a macro lens AF-S MICRO NIKKOR 105 mm 1:2.8G ED, a remote control, and three macro spacer rings with length of 12, 20, and 36 mm were used to take the pictures. For the lighting of the investigated area 2× Manfrotto ML360HP MIDI-36 (420LX@1m) were used.

**Figure 8:**
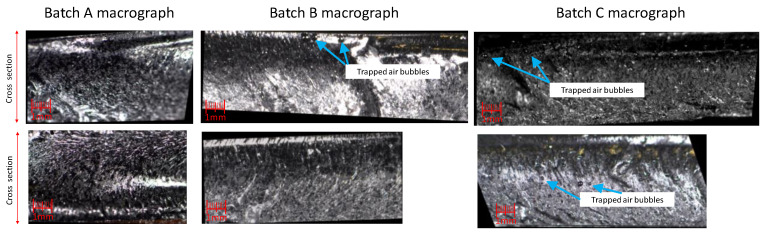
Macrographs of the cross-section of the Batch A (on the **left**), Batch B (on the **center**), and Batch C (on the **right**).

Looking at Figure 8, it is possible to notice some air bubbles trapped on the epoxy matrix of Batches B and C, which are not visible in Batch A. The air bubbles spotted on Batches B and C were probably generated by the longer mixture time in the third step of the manufacturing process (5 min of Batches B and C compared to 1 min of Batch A).

**Figure 9:**
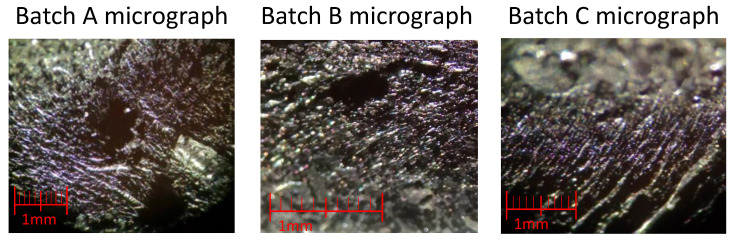
Micrographs of the cross-section of Batch A (on the **left**), Batch B (**center**), and Batch C (on the **right**).

Figure 9 shows micrographs obtained with an optical microscope, Leica MZ12 (Leica Microsystems, Wetzlar, Germany), of the cross section of lots A, B, and C. Holes, probably due to the presence of CNTs bundles, were found in both Batch A and Batch B, although in the latter they are smaller. It is not possible to identify such holes in Batch C at these magnification levels. These holes are not air bubbles since, as can be seen in Figure 8, they are spherical in shape.

As highlighted in paragraph 2.1, a total of 28 samples were tested with the proposed technique and all gave results consistent with those presented.

## 4. Discussion

The distinct thermal response to heat transfer transients of nanocomposites with different levels of dispersion can be explained taking into account the following considerations.

The physical phenomenon is primarily determined by the difference in the thermal conductivity of the epoxy resin, the MWCNTs bundles, and the individual MWCNTs, as reported in Table 2.

The individual MWCNTs have a much higher thermal conductivity than that found within the MWCNTs beams [37,38,39]. For the thermal conductivity of single-walled carbon nanotubes (SWNT), theoretical calculations predict very high values κ = 6600 W m^−1^ K^−1^ [40], but the experimental results [37,38,39] indicate that their thermal conductivity is much lower, particularly for single MWCNTs it is 600 ± 100 W m^−1^ K^−1^. The experiments also showed that within the bundles of MWCNTs the thermal conductivity is further reduced to 150 ± 15 W m^−1^ K^−1^ [37,38,39]. A study [37] explained this result with the substantial reduction in the transport abilities inherent for individual CNTs due to the quenching of phonon modes in bundles, reinforced by radial deformation of CNTs by van der Waals forces. The epoxy resin has a thermal conductivity almost two thousand times lower than that of MWCNTs and five hundred times lower than that of MWCNTs bundles. 

The presence of MWCNTs in the matrix has significant effects on the thermal response of the composites, this is an important aspect for the following discussion. The interface area between the MWCNTs and the matrix is maximized in the presence of a homogeneous dispersion, and so are the effects produced by the presence of MWCNTs on the thermal response of the composites. For example, for the same heating and cooling cycle, an addition of 0.5 wt % of MWCNTs makes the maximum temperature recorded by a thermographic analysis about 10 °C higher than in the case of pure epoxy resin, e.g., [41].

When the thermal waves generated by the external pulse propagate into the nanocomposite, the MWCNTs bundles clearly stand out in the phasegrams, since their thermal conductivity is much higher. Thus, their response is quicker than the epoxy resin, even when individual MWCNTs are well dispersed in the vicinity of the bundle. Moreover, when several bundles of MWCNTs are present, there are fewer single MWCNTs that are homogeneously dispersed in the epoxy resin, making the overall thermal conductivity of the composite epoxy-MWCNTs surrounding the bundle lower than it would be in a composite where the MWCNTs are all well dispersed. This latter effect further enhances the difference in thermal response seen with the PPT technique between the bundle and its surroundings environment, with respect to what can be seen in a composite where bundles are few and small. 

The effects of MWCNTs dispersion on the thermal response of nanocomposites are also attributable to a second mechanism: the specific heat of MWCNTs bundles is 50% higher than that of single MWCNTs and 25% lower than that of epoxy resin. So, a volume fraction of MWCNTs bundles will require less heat to raise the temperature, compared to the same volume fraction of the epoxy, even when the individual MWCNTs are well dispersed in the matrix.

In relation to the few air bubbles seen in the macrography shown in Figure 7, it must be said that since the thermal conductivity of the air is extremely small, around 0.0257 W/m K at 20 °C, the infrared camera could detect them. If the bubbles were present in the samples with coarse dispersion, there could be a doubt that the areas with different thermal response, which develop in a different phase, are due to the bubbles instead of the bundles. This was not the case, as can be seen in Figure 7 where the trapped bubbles are only in Batch C, demonstrating that the thermographic approach presented is not influenced by the presence of small air bubbles. 

The potential limitations of the proposed approach are as follows. The application of the technique requires a fast cooled IR-camera with a high sampling rate in order to captures the thermal transient. These IR-cameras can be expensive, especially if high definition and thermal contrast (NETD) are needed. Reflective coatings would greatly reduce the infrared radiation captured by the IR-Camera, reducing the signal to noise (S/N) ratio and the accuracy of the inspection. The probing capability of the technique (in the thickness direction) is limited by the power of the heat source, and by the conductivity and heat capacity of the sample.

## 5. Conclusions

In a previous work [29], a novel NDE technique based on infrared thermography, able to test the dispersion of the added nanoparticles in nanocomposites, was presented. The NDE technique was used to compare pairs of samples, and a significant difference in the thermal response to heat transfer transients was found. The method, however, was born with a crucial limitation—it works only as a comparison between pairs of specimens side by side, examined simultaneously by the IR-camera. Furthermore, the only difference between the two specimens must be the dispersion, and there must be no other difference either in terms of size, or in terms of composition.

To overcome this limitation, here an active infrared thermography NDT (IR-NDT) inspection technique was applied for the first time to epoxy-CNTs composites, to evaluate the level of dispersion of the nanoparticles. Infrared-NDT techniques (IR-NDT) allow fast full-field non-contact inspections of large areas, with relatively inexpensive and flexible hardware setups. The pulsed phase thermography (PPT) approach was tested on three types of epoxy nanocomposite samples with dissimilar degrees of nanoparticle dispersion prepared with different procedures. The phasegrams obtained with this technique clearly show clusters, or bundles, of CNTs when present, so a comparison with the reference sample is not necessary to evaluate the quality of the dispersion as for the previous approach. 

Thus, the novel NDE technique can be used to check the quality, in terms of dispersion levels, of products made of nanocomposites based on epoxy and MWCNTs, in order to guarantee that the expected specifications are met.

The mechanisms behind the effects of MWCNTs dispersion on the thermal response of the nanocomposites were identified.

## Figures and Tables

**Table 1:** Properties of the bi-component epoxy resin and of the Multi-wall Carbon Nanotubes.

**Specification of the Epoxy Resin**	
Brand/Product	MATES/SX 8 EVO
Type	Bisphenol A modified Epoxy resin
Physical state	Liquid
Gardner index	3
Viscosity at 25 °C	500 ÷ 600 mPas
Specific weight at 20 °C	1.14 ÷ 1.16 g/cm^3^
Flash point	100 °C
**Specification of the Hardener**	
Type	modified cycloaliphatic polyamine
Physical state	Liquid
Gel time at 25 °C	2/4 h
Flash point	190 °C
Mixing ratio (epoxy:hardner)	10:3
**Specification of the Carbon Nanotubes**	
Type	Multi-Wall Carbon Nanotubes
Outer diameter	20–30 nm
Length	~50 µm
Purity	>95 wt %
Ash	<1.5 wt %

**Table 2:** Thermal properties of the materials.

Properties	Epoxy	Air	MWCNTs Single	MWCNTs in Bundle
Thermal conductivity [W/m K]	0.35	0.026	600 ± 100	150
Specific heat capacity [J/kg K]	1000	1000	500	750
Density [kg/m^3^]	1150 ± 10	1.22	2600	-

**Table 3:** Pulsed Thermography setup and Pulsed Phase algorithm parameters.

**Experimental Parameters**	
Distance sample to IR camera	~71 cm
Distance flash lamp to sample surface	~65 cm
Sample rate IR camera	165 Hz
Integration time	3000 µs
**Pulsed Phase Thermography Parameters**	
Truncation window	500 frames
Sub-window dimensions [pixel × pixel]	~240 × 260
